# Increased levels of (class switched) memory B cells in peripheral blood of current smokers

**DOI:** 10.1186/1465-9921-10-108

**Published:** 2009-11-12

**Authors:** Corry-Anke Brandsma, Machteld N Hylkema, Marie Geerlings, Wouter H van Geffen, Dirkje S Postma, Wim Timens, Huib AM Kerstjens

**Affiliations:** 1 Department of Pulmonary Diseases, University Medical Center Groningen, University of Groningen, P.O. Box 30.001, 9700 RB, Groningen, The Netherlands; 2 Department of Pathology, University Medical Center Groningen, University of Groningen, P.O. Box 30.001, 9700 RB, Groningen, The Netherlands

## Abstract

There is increasing evidence that a specific immune response contributes to the pathogenesis of COPD. B-cell follicles are present in lung tissue and increased anti-elastin titers have been found in plasma of COPD patients. Additionally, regulatory T cells (Tregs) have been implicated in its pathogenesis as they control immunological reactions. We hypothesize that the specific immune response in COPD is smoke induced, either by a direct effect of smoking or as a result of smoke-induced lung tissue destruction (i.e. formation of neo-epitopes or auto antigens). Furthermore, we propose that Tregs are involved in the suppression of this smoke-induced specific immune response.

The presence of B cells, memory B cells and Tregs was assessed by flow cytometry in peripheral blood of 20 COPD patients and 29 healthy individuals and related to their current smoking status.

COPD patients had lower (memory) B-cell percentages and higher Treg percentages in peripheral blood than healthy individuals, with a significant negative correlation between these cells. Interestingly, current smokers had higher percentages of (class-switched) memory B cells than ex-smokers and never smokers, irrespective of COPD.

This increase in (class-switched) memory B cells in current smokers is intriguing and suggests that smoke-induced neo-antigens may be constantly induced in the lung. The negative correlation between B cells and Tregs in blood is in line with previously published observations that Tregs can suppress B cells. Future studies focusing on the presence of these (class switched) memory B cells in the lung, their antigen specificity and their interaction with Tregs are necessary to further elucidate the specific B-cell response in COPD.

## Introduction

COPD is a leading cause of death worldwide and its morbidity and mortality are still rising. Although the pathogenesis of the disease is still not fully defined, tobacco smoke is widely accepted as the most important cause for the development of the disease certainly in the western world. Until now, the only effective treatment to stop the accelerated lung function decline is smoking cessation, even though the inflammatory response may persist [1]. More information is needed about the origins and nature of the chronic inflammatory response in COPD to find better treatment targets for COPD patients.

The role of the innate immune response, i.e. neutrophils and macrophages is well established in COPD, as is the role of CD8 T cells [2,3]. Yet the role of other important cells in specific immunity, in particular CD4 T cells and B cells, have only recently attracted attention. We and others have found both oligoclonal T- and B cells in the lungs of COPD patients suggesting an antigen driven immune response [4,5]. Furthermore, Lee et al recently demonstrated a specific Th1 response against lung elastin in patients with emphysema [6]. Additionally, an increased number of small airways containing B cells and lymphoid follicles has been shown in patients with GOLD stage III-IV compared to stage 0-II [7], as well as an increase of B cells in the mucosa of large airways in COPD patients compared to controls [8]. At present it is largely unclear against which antigen(s) this specific immune response in the lungs of COPD patients is directed. In this respect, at least three potential sources of antigens should be considered: 1) microbial, 2) cigarette smoke components or derivatives, and 3) auto-antigens, encompassing (neo) antigens derived from degradation products of extracellular matrix. The latter is supported by the recent findings regarding an immune response against elastin [6] and the presence of anti nuclear auto-antibodies in COPD [9].

An important modulator of the immune system is the regulatory T cell (Treg). Tregs express CD4, CD25 and forkhead transcription factor 3 (Foxp3) and are important in controlling immunological tolerance and preventing auto-immune reactions by inhibiting T-cell responses [10]. In addition, Tregs can directly inhibit B-cell responses by suppressing class switch recombination and Ig production [11,12]. Given this link between Tregs and B cells, it is tempting to speculate about a diminished role for Tregs in the suppression of the specific B-cell response in COPD.

So far, only four studies have investigated the presence of Tregs in COPD, but they reported different findings in lung tissue and bronchoalveolar lavage (BAL). First, decreased numbers of CD4^+^CD25^+ ^Tregs and Foxp3 mRNA levels were shown in lung tissue of emphysema patients compared to control subjects [6]. Additionally, increased numbers of CD4^+^CD25^bright ^Tregs were shown in BAL from COPD patients and healthy smokers compared to healthy never smokers [13], while another group showed decreased CD4^+^CD25^+ ^Tregs in BAL of COPD patients and never smokers compared to healthy smokers [14]. Finally, an immunohistochemical study demonstrated increased numbers of Foxp3^+ ^cells in large airways of asymptomatic smokers and COPD patients compared to non-smokers, and decreased numbers of Foxp3^+ ^cells in small airways of COPD patients compared to asymptomatic smokers and non-smokers [15].

We hypothesize that the specific immune response in COPD is smoke induced and is either a direct result of smoking or a result of the smoke-induced lung tissue destruction (i.e. formation of neo-epitopes or auto antigens). We propose that Tregs are involved in the suppression of this smoke induced specific immune response and that a diminished presence or function on these cells may underlie the development of the specific humoral immune response in COPD.

We investigated the presence of B cells, memory B cells, and Tregs in peripheral blood obtained from smoking and ex-smoking COPD patients and smoking, ex-smoking and never-smoking healthy volunteers.

## Methods

### Subjects

COPD patients and healthy individuals were recruited to participate in this study. Inclusion criteria for COPD patients were; clinical diagnosis of COPD, post bronchodilator FEV_1 _< 80% predicted, post bronchodilator FEV_1_/FVC < 70%, and no exacerbation in the 6 weeks preceding the study. Inclusion criteria for healthy individuals were; no signs and symptoms of pulmonary disease, FEV_1 _> 90% predicted, and FEV_1_/FVC > 70%.

All participants met the following criteria: age > 40 years, negative skin prick tests for the most common aeroallergens, no use of (inhaled or systemic) corticosteroids in the 6 weeks preceding the study, and no major co morbidities. To avoid the effect of gender only males were included in the study. Smokers and ex-smokers had to have a smoking history of at least 10 packyears and ex-smokers had to have quit smoking for a least one year. The medical ethics committee of the University Medical Center Groningen approved the study and all participants gave their written informed consent.

### Cell isolation

All participants donated 20 ml of peripheral blood. Peripheral blood mononuclear cells (PBMCs) were isolated using ficoll-paque plus (GE Healthcare, UK) density gradient centrifugation. Total isolated cells were counted using a Sysmex pocH-100i cell counter (Sysmex, Roche, Germany). Cells were used for flow cytometry and immunocytochemical staining on cytospins.

### Flow cytometry analysis

Two antibody cocktails were used to stain PBMCs for 1) B cells and 2) Tregs.

1. CD20-PE-Cy5, CD27-FITC, and IgM-biotin followed by Streptavidin-PE (all BD Biosciences).

2. CD4-AmCyan (BD Biosciences, San Jose, USA), CD25-Pe-Cy7 (eBioscience, San Diego, USA) and Foxp3-Alexa Fluor 700 (eBioscience).

Appropriate isotype controls were used for the CD25 (mouse IgG1-Pe-Cy7, eBioscience) and Foxp3 (rat IgG2a-Alexa Fluor 700, eBioscience) staining.

Before staining the surface markers, 10^6 ^cells per 25 μl were first incubated for 15 minutes on ice with cold 0.5% human serum (Sigma-Aldrich, Zwijndrecht, the Netherlands) to block a-specific binding sites. Plates were centrifuged and cells were subsequently incubated with the appropriate antibody cocktail for 30 minutes on ice, protected from light. After washing the cells of both cocktails with phosphate buffered saline solution (PBS) supplemented with 2% bovine serum albumin (BSA, Serva, Heidelberg, Germany), the cells of cocktail 1 were incubated for 15 minutes with Streptavidin-PE, washed three times with PBS/2%BSA, resuspended in FACS lysing solution (BD Biosciences), and kept in the dark on ice until flow cytometry analysis. The cells of cocktail 2 were fixed and permeabilized for 30 minutes using a fixation and permeabilization buffer kit (eBioscience), and then washed with permeabilization buffer, blocked with 2% human serum and then incubated with anti-Foxp3 for 1 hour. Afterwards the cells were washed with permeabilization buffer, resuspended in FACS lysing solution, and kept in the dark on ice until flow cytometric analysis. The fluorescent staining of the cells was measured on a LSR-II flow cytometer (BD Biosciences) and data were analyzed using FlowJo Software (Tree Star, Ashland, USA).

**Figure 1:**
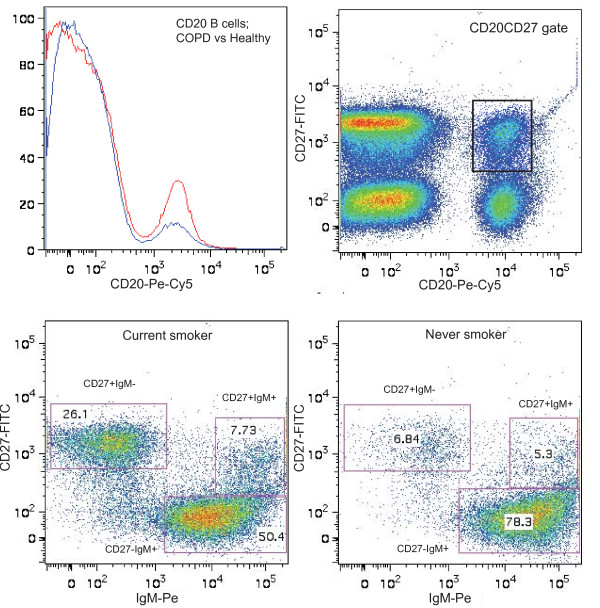
**Flow cytometry plots of B cells and memory B cells in peripheral blood**. A representative example of the difference in percentage of CD20^+ ^B cells between COPD (blue curve) and healthy (red curve) and the CD20^+^CD27^+ ^gate to analyze the memory B cells is depicted in the upper panel. The CD27^+^IgM^- ^gate for class switched memory B cells, the CD27^+^IgM^+ ^gate for IgM^+ ^memory B cells, and the CD27^-^IgM^+ ^gate for naive B cells are shown for a current and a never smoker in the lower panel.

Based on the expression of CD20, CD27, and membrane IgM, different B-cell subsets were distinguished. Within the lymphocyte gate, total B cells were analyzed based on CD20 expression, and total memory B cells were analyzed based on co-expression of CD20 and CD27 (Figure 1). Within the CD20 population, naive B cells (CD27^-^IgM^+^), IgM^+ ^memory B cells (CD27^+^IgM^+^), and class-switched memory B cells (CD27^+^IgM^-^) were distinguished.

**Figure 2:**
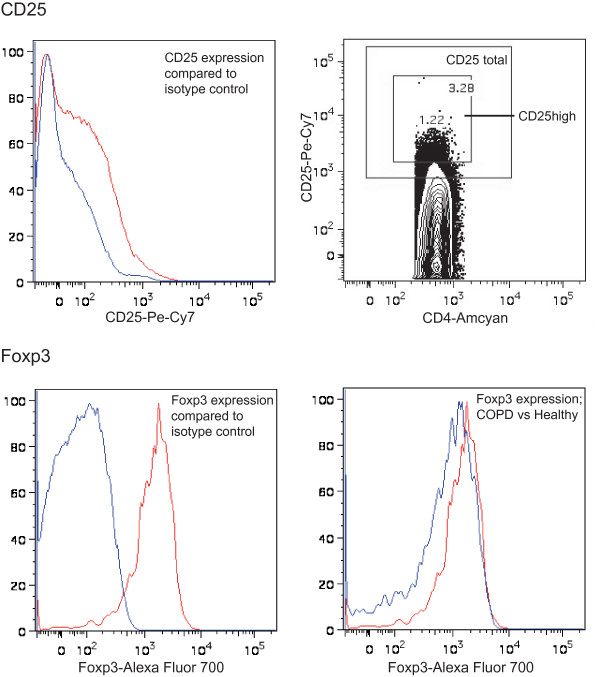
**Flow cytometry plots of regulatory T cells in peripheral blood**. The CD25 expression (red curve) compared to the isotype (blue curve), and the CD25 total and CD25high gates are depicted in the upper panel. The Foxp3 expression (red curve) compared to the isotype (blue curve), and an example of the difference in Foxp3 expression between COPD (red curve) and healthy (blue curve) are depicted in the lower panel.

Tregs were defined as CD4^+^CD25^+^Foxp3^+ ^T cells. The positive gates for CD25 and Foxp3 expression were based on the expression levels of the appropriate isotype controls, and a separate CD25^high ^gate was set on the high population (Figure 2).

### Immunocytochemistry

The presence of cells expressing the different Ig isotypes IgE, IgG and IgA was assessed using immunocytochemical staining of PBMC cytospins. IgE, IgG and IgA expression was demonstrated by a rabbit-anti IgE antibody (Dako, Heverlee, Belgium) followed by a biotin labeled goat-anti-rabbit secondary antibody (SBA, Birmingham, USA) and AB complex (Dako), a direct labeled anti-IgG-Fitc antibody (Protos Immunoresearch, Burlingame, USA), and an anti-IgA (Dako) antibody followed by a biotin labeled rabbit-anti-mouse secondary antibody (Dako) and AB complex, respectively. Per cytospin, 600 cells were counted and expressed as percentage positive cells.

### Statistical analysis

A multiple linear regression model was used to determine whether the levels of B cells, memory B cells and Tregs differed by current smoking status or by having COPD or their combination. This method disentangles the separate effects of COPD and current smoking and their interaction. First, the effects of COPD and current smoking were tested together with the interaction between COPD and current smoking as independent variables. When the interaction between COPD and current smoking was not significant, the effects of COPD and current smoking were tested again without the interaction term. The normal distribution of the residuals was analyzed with a Kolmogorov-Smirnov test and when needed the data were log-transformed to normalize distributions. Additionally, Mann Whitney U tests were used to establish differences between all the subgroups according to the presence of COPD and the current smoking status. The relation between B cells and CD4^+^CD25^+^Foxp3^+ ^T cells, and (class-switched) memory B cells and IgA expression was evaluated with the Spearman correlation. A value of p < 0.05 was considered significant.

## Results

### Patient characteristics

The characteristics of the twenty COPD patients (current and ex-smokers) and twenty-nine healthy volunteers (current, ex- and never smokers), included in the study, are shown in table 1. Healthy individuals were slightly younger than the COPD patients, which was mainly caused by the young age of the healthy smokers. Additionally, COPD patients had more packyears of smoking when compared to healthy current and ex-smokers. One healthy person was included as "never smoker" who had a smoking history of 2.5 packyears and had stopped smoking for 40 years, the other never smokers had no smoking history at all.

**Table 1:** Characteristics of COPD patients and healthy individuals

	COPD patients		Healthy individuals		
	Current smokers	Ex-smokers	Current smokers	Ex-smokers	Never smokers
Subjects (n)	10	10	9	10	10

Age (years)	65.9 (4.3) *	66.7 (7.4) *	52.8 (4.1) ^#^	61.1 (9.3)	58.1 (6.5)

Packyears	34 (13.5) *	36.7 (18.2) *	24.6 (11)	20.6 (5.9)	0.3 (0.8)

FEV_1 _post BD (% pred.)	44.9 (14.9)^$^	60.7 (14.7)	105.6 (8.7)	115.7 (15.9)	111.1 (12.1)

FEV_1_/FVC post BD (%)	37.6 (10)	43.8 (10.7)	76.4 (4)	78.2 (5.9)	78.5 (4.1)

Mean (standard deviation) is depicted. Mann Whitney U tests were used to test differences between the groups. FEV_1 _= Forced expiratory volume in 1 second. FVC = Forced vital capacity. BD = Bronchodilator.

* COPD patients versus healthy individuals; packyears p = 0.006, age p = 0.000

^# ^Healthy current smokers versus healthy ex-smokers p = 0.01 and versus healthy never smokers p = 0.045

^$ ^COPD smokers versus COPD ex-smokers p = 0.03

### B cells, memory B cells, and Ig isotypes in peripheral blood

#### COPD versus healthy

**Figure 3:**
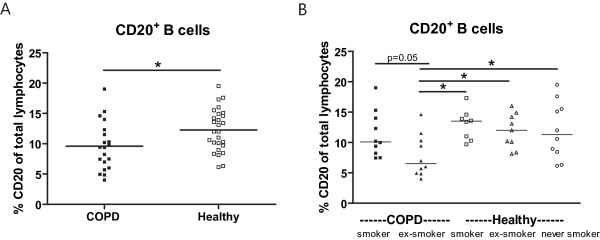
**B cells in peripheral blood**. A) Percentages of total B cells in peripheral blood of COPD patients (closed symbols) and healthy individuals (open symbols). The result of the multiple linear regression analysis (i.e. corrected for current smoking) is depicted in the figure. B) The same results are depicted, but divided in subgroups based on the presence of COPD and the current smoking status. In this figure the results of the Mann Whitney U tests are depicted. * indicates that p < 0.05

**Figure 4:**
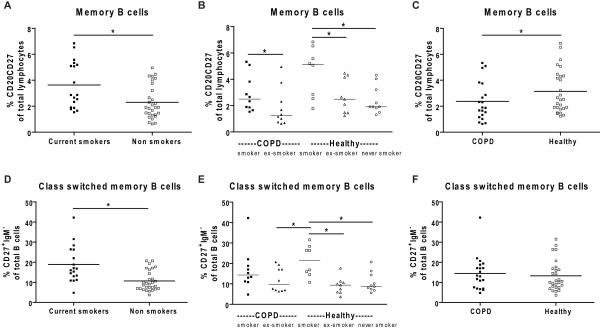
**Memory B cells in peripheral blood**. A) Percentages of memory B cells and class switched memory B cells (D) in peripheral blood of current smokers (closed symbols) and non smokers (open symbols). In B), C) and E), F) the same results are depicted, but divided into subgroups based on the current smoking status and COPD versus healthy controls. In A) and D) the results of the multiple linear regression analysis corrected for having COPD are depicted. In C) and F) the results of the multiple linear regression analysis corrected for smoking are depicted. In B) and E) the results of the Mann Whitney U tests are depicted. * indicates that p < 0.05

**Figure 5:**
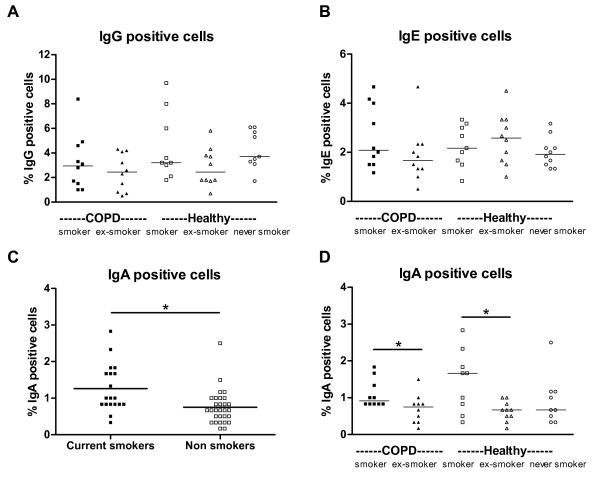
**IgG, IgE and IgA positive cells in peripheral blood**. Percentages of A) IgG, B) IgE and D) IgA positive cells in peripheral blood of COPD patients (closed symbols) and healthy individuals (open symbols) are depicted and divided in subgroups based on the presence of COPD and the current smoking status. The results of the Mann Whitney U tests are depicted in these figures. In C) the same results for IgA are depicted, but divided in current smokers (closed symbols) and non-smokers (open symbols). In this figure the result of the multiple linear regression analysis (i.e. corrected for having COPD) is depicted. * indicates that p < 0.05

COPD patients had lower percentages of total B cells (p = 0.006, Figure 3A) and memory B cells (p = 0.004, Figure 4) compared to healthy individuals. There was a similar trend (p = 0.08, Figure 5A) for IgG positive cells. No differences were found between COPD patients and healthy controls with respect to numbers of IgA and IgE positive cells (Figure 5).

When analyzing the groups based on their current smoking status, COPD ex-smokers had lower B-cell percentages than healthy smokers (p = 0.01), ex-smokers (p = 0.02) and never smokers (p = 0.03) and a trend (p = 0.05) when compared to COPD smokers (Figure 3B).

The lower percentages of B cells in COPD could not be explained by the difference in age or packyears between COPD patients and healthy individuals (p > 0.05, when age or packyears was added to the multiple regression analysis).

#### Effect of current smoking

Current smokers (COPD and healthy combined) had higher percentages of memory B cells (p < 0.001, Figure 4A) and class-switched memory B cells (p < 0.001, Figure 4C) than ex-smokers and never smokers (combined). There was a similar trend for total B cells (p = 0.05, Figure 3).

When analyzing the groups based on their current smoking status, COPD smokers had higher percentages of memory B cells than COPD ex-smokers (p = 0.03, Figure 4B). Also within healthy individuals, current smokers had higher percentages of memory B cells than ex-smokers (p = 0.03) and never smokers (p = 0.02). Similar results were present for class switched memory B cells; healthy smokers had higher percentages of class-switched memory B cells than healthy ex-smokers (p = 0.002) and never smokers (p = 0.003, Figure 4D).

The expression of the different Ig subtypes was analyzed on PBMC cytospins to asses to which isotype the memory B cells had switched. Current smokers (COPD and healthy combined) had more IgA positive cells than ex- and never smokers (p = 0.002, Figure 5C). This current smoking effect was not present for IgE and IgG positive cells.

**Figure 6:**
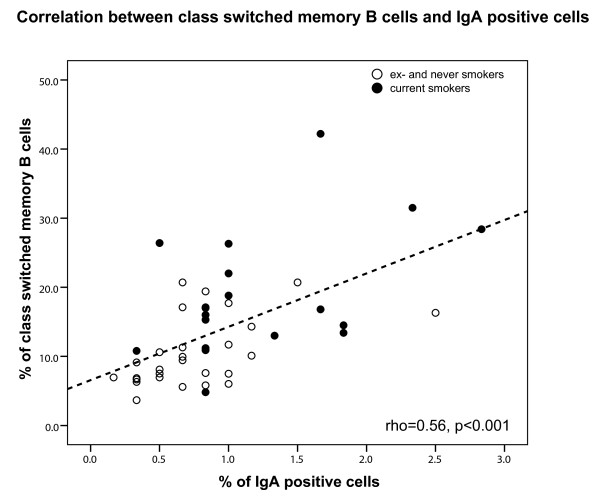
**Correlation between class switched memory B cells and IgA positive cells**. Correlation between class switched memory B cells and IgA positive cells for current smokers (black circles) and ex-and never smokers (open circles). The result of the Spearman correlation is depicted in the figure.

When analyzing the groups based on their current smoking status, COPD smokers had higher percentages of IgA positive cells than COPD ex-smokers (p = 0.03, Figure 5D). Also within healthy individuals, current smokers had higher percentages of IgA positive cells than ex-smokers (p = 0.03). Furthermore, the percentages of IgA positive cells were positively correlated with memory B cells (rho = 0.46, p = 0.001) and class switched memory B cells (rho = 0.56, p < 0.001, Figure 6).

There were no effects of COPD or current smoking on IgM^+ ^memory B cells and naive B cells (data not shown).

### Regulatory T cells in peripheral blood

#### COPD versus healthy

**Figure 7:**
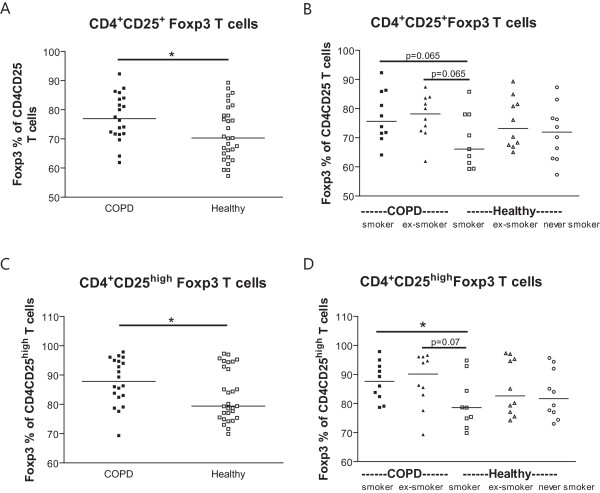
**Regulatory T cells in peripheral blood**. A) Foxp3 percentages of CD4^+^CD25^+ ^T cells and C) CD4^+^CD25^high ^T cells in peripheral blood of COPD patients (closed symbols) and healthy individuals (open symbols). The results of the multiple linear regression analysis (i.e. corrected for current smoking) are depicted in the figures. In B) and D) the same results are depicted, but divided in subgroups based on the presence of COPD and the current smoking status. In these figures the results of the Mann Whitney U tests are depicted. * indicates that p < 0.05

COPD patients had higher percentages of CD4^+^CD25^+^Foxp3^+^T cells (p = 0.03, Figure 7A) and CD4^+^CD25^high ^Foxp3^+^T cells (p = 0.04, Figure 7C) than healthy individuals.

When analyzing the groups based on their current smoking status, COPD smokers had a higher percentage of CD4^+^CD25^high^Foxp3^+ ^T cells than healthy smokers (p = 0.049, Figure 7D), which was also true for the CD4^+^CD25^+^Foxp3^+ ^T cells (trend (p = 0.065), Figure 7B). No differences were found between COPD and healthy individuals with respect to CD4 T cells, CD4^+^CD25^+ ^T cells, and CD4^+^CD25^high ^T cells (data not shown).

The differences in percentages of CD4^+^CD25^+^Foxp3^+^T cells could not be explained by the difference in age or packyears of smoking between COPD patients and healthy individuals (p > 0.05, when age or packyears was added to the multiple regression analysis).

#### Effect of current smoking

There were no effects of current smoking with respect to CD4 T cells, CD4^+^CD25^+ ^T cells, CD4^+^CD25^high ^T cells and CD4^+^CD25^+^Foxp3^+^T cells in peripheral blood.

### Correlation between regulatory T cells and B cells

**Figure 8:**
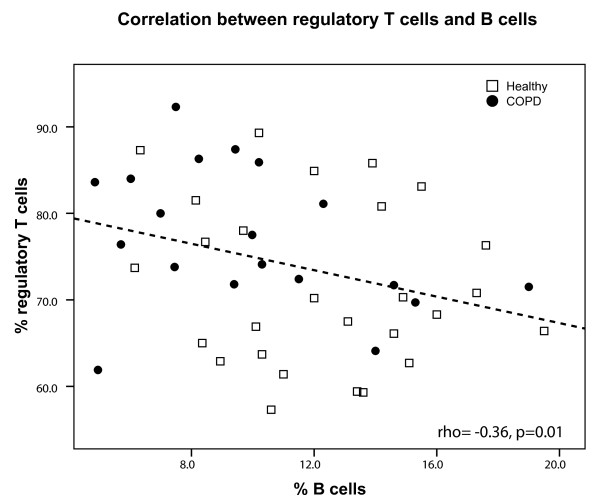
**Correlation between regulatory T cells and B cells**. Correlation between CD4^+^CD25^+^Foxp3^+ ^T cells and total B cells for COPD patients (black circles) and healthy individuals (open squares). The result of the Spearman correlation is depicted in the figure.

The percentage of CD4^+^CD25^+^Foxp^+ ^T cells was negatively correlated with the percentage of B cells (rho = -0.36, p = 0.01, Figure 8) and memory B cells (rho = -0.34, p = 0.02). For COPD alone, the correlation between CD4^+^CD25^+^Foxp^+^T cells and B cells was of the same magnitude, but due to less power it did not reach statistical significance (rho = -0.40, p = 0.08).

## Discussion

In this study we had two main observations. First, patients with COPD had lower percentages of (memory) B cells and higher percentages of Tregs in peripheral blood compared to healthy individuals. These higher Treg percentages correlated significantly with both lower total B cell and memory B cell percentages. Second, current smokers had higher percentages of total memory B cells as well as class-switched memory B cells in peripheral blood, regardless of the disease state. Additional Ig subtype analysis suggested that this increased class switched memory B cell population consists mainly of IgA expressing B cells.

In addition to our previous studies in which B cells were studied in lung tissue of COPD patients [4,8], we now have studied the presence of B cells and memory B cells in peripheral blood of COPD patients and healthy individuals. Except for one earlier publication from our group that showed decreased total B-cell percentages in COPD non-smokers compared to COPD smokers [16], we could not find any data assessing the presence of B-cells and memory B cells in peripheral blood of patients with COPD. With respect to our first main observation, the lowest B-cell percentages were detected in the COPD ex-smokers, consistent with the earlier findings of de Jong *et al. *[16]. Although speculative, the decreased percentage of total B cells in peripheral blood of COPD patients and the previously described increased presence of B cells in lung tissue of COPD patients [7,8] could reflect an increased recruitment of B cells from the periphery to the lung, perhaps related to increased presence of antigens in the lungs. Since B cells were expressed as the percentage of total lymphocytes, we can not exclude that the decreased percentage of B cells in COPD patients may be related to an increased percentage of CD8 cells, which was already demonstrated in COPD before [16,17].

Regarding our second main observation, current smokers had significantly more memory B cells including class-switched memory B cells than ex- and never smokers. This is intriguing since class-switched memory B cells are mature B cells that have replaced their primary encoded membrane receptor (IgM) by IgG, IgA or IgE in response to repeated antigen recognition [18]. This process of class-switch recombination is mostly dependent on the presence of specific antigen-antibody complexes in germinal centers (GC), and thus the extent of this GC mediated level of class-switching is related to actual presence of antigen and recognizing antibody. Therefore, the finding of increased class-switched memory B cells in our current smokers suggests the possibility of a chronic antigen-specific immune response that is particularly caused by ongoing smoke-induced formation or release of (neo)-antigens (e.g. matrix degradation products or smoke particles). The primary immune response to these antigens may be weak, but may still lead to the formation of memory B cells. When the antigen stimulus (tobacco smoke) is present for a prolonged period, secondary immune responses may lead to increased numbers of memory B cells and plasma cells, and a continued presence of memory B cells, as shown in the current smokers in our study.

Because this increase in memory B cells is only present in the current smokers and does not distinguish between COPD patients and healthy controls, one might argue whether it is important for COPD pathogenesis. We speculate that this specific immune response is to a certain extent present in all smokers with a considerable smoking history and is not the only factor leading to COPD pathogenesis. Other important factors like the underlying genetic predisposition, in combination with environmental factors may contribute to a large extent to the development of the chronic inflammatory response and emphysema development, which distinguishes COPD patients from asymptomatic smokers. Genetic predisposition can have profound effects on many immunologic processes, including the lack of immune suppression by Tregs.

As mentioned in the introduction, the presence of CD4^+^CD25^+ ^Tregs in COPD has been investigated previously [6,13-15]. These studies reported different, partially contradictory, findings in lung tissue and bronchoalveolar lavage, and reported no differences in CD4^+^CD25^+ ^Tregs in peripheral blood between COPD patients and healthy controls. However, in these studies, the presence of Tregs was analyzed by measuring CD4^+^CD25^+ ^T cells. Foxp3 expression in these cells was assessed in separate analyses to prove that a high percentage of these CD4^+^CD25^+ ^T cells were positive for Foxp3 and thus Tregs. Instead, we analyzed Tregs by measuring the percentage of Foxp3 expressing CD4^+^CD25^+ ^T cells and with this method increased Treg percentages in peripheral blood of COPD patients were found when compared to healthy individuals. In our view, the way of identifying Tregs explains the discrepant findings between the previous studies and our study. This is supported by the fact that with a similar analysis compared to the previous studies we could also not detect differences in CD4^+^CD25^+ ^or CD4^+^CD25^high ^T cells between COPD patients and healthy individuals.

Nevertheless, the observation that the percentage of Tregs and the level of Foxp3mRNA is decreased in lung tissue of COPD patients [6] together with the increased percentage of Tregs in peripheral blood in our study could suggest a decreased infiltration of Tregs to the lung in COPD. Together with the increased B cell numbers in lung tissue this might represent a local imbalance between B cells and Tregs in the lung. Unfortunately there is no data yet analyzing the balance between Tregs and B cells in lung tissue. The only data supporting a relation between Tregs and B cells in the lung comes from our smoking mouse model, in which we showed a relation between the levels of Foxp3 positive cells and the number of B cell infiltrates in lung tissue [19].

We assessed the presence of Tregs and B cells in peripheral blood and it can be argued whether this gives a good reflection of the inflammatory response in the lungs. Animal data showed that BAL lymphocytes can migrate to regional lymph nodes and recirculate in the blood [20]. However, several studies investigating Tregs in different compartments, i.e. BAL, lung tissue and blood, showed discrepant findings in the different compartments comparing COPD and healthy controls [6,13,14]. Furthermore, it is known that the inflammatory environment, particularly high levels of TNF-α, affects the Foxp3 expression and functionality of Tregs [21]. Thus, in order to draw conclusions about a possible role for Tregs in COPD, the crucial next step is to study the presence and particularly the functionality of local Tregs in the lung.

With respect to the B cells, this study showed that smoking can lead to increased levels of circulating memory B cells. Given the fact that B cells traffic to the circulation after antigen recognition in the lung, this smoke induced memory B-cell response in blood could very well be a reflection of the specific B-cell response in the lung. This is supported by our observation that the increased class switched memory B cell population consists mainly of IgA expressing B cells, reflecting a mucosal immune response.

In conclusion, we showed that smoking may induce a specific immune response, which is reflected by increased percentages of circulating (class switched) memory B cells. We propose that a smoke-induced specific immune response is involved in the chronic inflammatory response in COPD. Future studies focusing on the presence of (class switched) memory B cells in the lung and their antigen specificity are necessary to further elucidate the specific B-cell response in COPD. Additionally, we showed increased percentages of circulating Tregs in COPD in association with decreased B cell percentages. These findings provide support for a relation between Tregs and B cells in COPD, which needs to be further explored in lung tissue. Preferably, Treg functionality in the lung should be related to parameters reflecting the specific B cell response in the lung.

## Competing interests

The authors declare that they have no competing interests.

## Authors' contributions

CB recruited the patients, analyzed the data, performed statistical analysis and drafted the manuscript. MH participated in the study design and data analysis, and helped to draft the manuscript. MG carried out the cell isolations, the flow cytometry analyses and was involved in the immunocytochemical analyses. WG performed the immunocytochemical stainings and analyses. WT and DS were involved in the study design and the patient recruitment, and critically reviewed the manuscript. HK participated in the study design, was supervisor of the patient recruitment, helped with the statistical analyses and critically reviewed the manuscript. All authors read and approved the final manuscript.

## Funding

This study was financially supported by the Graduate School for Drug Exploration (GUIDE) of the University of Groningen and the Dutch Asthma Foundation.
